# Estimating country-level spending on pandemic preparedness

**DOI:** 10.7189/jogh.14.03026

**Published:** 2024-06-17

**Authors:** Mukesh Chawla, Rocio Schmunis

**Affiliations:** World Bank, Washington D.C., USA

## THE CONTEXT

Stunned by the profound human and economic toll caused by the global coronavirus disease 2019 (COVID-19) pandemic and the grim foreboding that this crisis might be a precursor to more frequent and severe pandemics, numerous commissions, task forces, conferences, and international discussions, convened in the wake of the pandemic, advocated for substantial investments to enhance national and regional capabilities for the early detection and containment of outbreaks. On 14 September 2020, the Global Preparedness Monitoring Board issued a report – A World in Disorder – which critically evaluates the global COVID-19 response and warns against unpreparedness for future pandemics [1]. On 20 April 2021, the UK inaugurated the International Pandemic Preparedness Partnership to ‘save lives from future diseases and prevent another global pandemic’ [2]. Subsequently, on 26 April 2021, the United Nations (UN) organised a high-level virtual dialogue – Pandemic Preparedness and Response Financing Architecture – to initiate discussions within the global health community towards consensus on establishing financing for sustainable health security [3]. Building on this momentum, the Global Health Summit, co-hosted by the European Commission and the Italian G20 presidency on 21 May 2021, resulted in the adoption of the Rome Declaration [4]. This document outlined principles and commitments to strengthen the existing multilateral health architecture for preparedness, prevention, detection, and response. On 9 July 2021, the G20 High-Level Independent Panel on Financing the Global Commons for Pandemic Preparedness and Response unveiled a report titled ‘A Global Deal for Our Pandemic Age’ urging swift action from the G20 and the international community to address shortfalls in the international COVID-19 response, estimated to be at least USD 75 billion over the next five years, or USD 15 billion per year [5].

Reminiscent of the flurry of similar initiatives following the 2014–15 West Africa Ebola outbreak, these discussions predominantly centred around the mobilisation of huge sums of money at every level of influence to prepare the world for the next morbific onslaught that any known or unknown infective agent may unleash. Some of these efforts have already achieved notable success, the most prominent being the establishment of the Pandemic Fund. Launched on 13 November 2022, on the margins of the G20 Joint Finance and Health Ministers’ meeting in Bali, the fund has initiated its first round of funding allocations amounting to USD 338 million to benefit 37 countries [6].

However, even as many low- and low-middle-income countries prepare to make a case for additional financing from the Pandemic Fund and other initiatives, few have baseline estimates of how much they spend on preparedness. Knowing how much countries spend annually on strengthening preparedness is an important initial step for understanding how existing funding resources have been used and the relative magnitude of spending change necessary to prevent the next global pandemic adequately. Highlighting areas where countries may be underinvesting will allow targeted interventions to strengthen these areas. Moreover, tracking and monitoring expenditures to ensure that funds are being used in line with strategic priorities and generating the desired outcomes will promote transparency and accountability and demonstrate effective management of resources – all of which would strengthen the case for additional donor support for strengthening preparedness.

There are two distinct but related reasons why most countries do not routinely measure spending on preparedness. First, the term preparedness encompasses various definitions depending on the context and use. It is difficult for countries to prescribe definitive bounds within which all elements of preparedness can be contained. Indeed, the International Health Regulations 2005, the purpose and scope of which is ‘to prevent, protect against, control and provide a public health response to the international spread of disease,’ does not even use the term preparedness, opting instead to refer to a broad range of public health aims and activities [7]. Second, most countries do not have pandemic preparedness as a spending category in the government budgets and associated reporting systems, which makes it challenging to identify, isolate and measure preparedness spending. In this article, we propose a practical way of overcoming these twin challenges and establishing mechanisms for routine estimation of preparedness spending.

## DEFINING PREPAREDNESS

**Figure 1:**
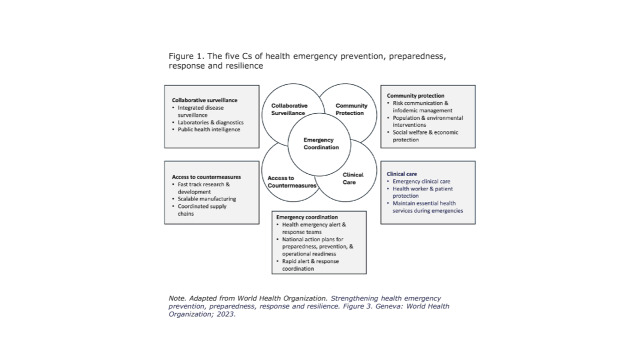
The five Cs of health emergency prevention, preparedness, response and resilience. **Table:** 

For the present exercise, we define preparedness expenditure as including all spending from all sources (public, private, external) on all activities whose primary purpose is assuring knowledge, capacities, capabilities (i.e. abilities to make good use of the capacities) and organisational structures in a country to prevent, detect, control, and provide an effective societal response to the spread of diseases that endanger population health within and across international boundaries. We propose defining the boundaries of preparedness by either the 19 technical areas in the World Health Organization’s (WHO) Joint External Evaluation (JEE) [8] or the five sub-systems emphasised in WHO’s latest guideline [9] (**Figure 1**, **Table 1**). Both approaches enjoy widespread acceptance, with the JEE tool in use since 2016 in multiple countries and the five sub-systems, though newer, currently undergoing implementation.

Table 1. Technical areas of the JEE tool (3rd edition)*Prevent	Detect	Respond	IHR-related hazards and points of entry
Legal documents	National laboratory system	Health emergency management	Points of entry and border control
Financing	Surveillance	Linking public health and security authorities	Chemical events
IHR coordination	Human resources	Health services provision	Radiation emergencies
Antimicrobial resistance		Infection prevention and control	
Zoonotic disease		Risk communication and community engagement	
Food safety			
Biosafety and biosecurity			
Immunisation			

IHR – International Health Regulations

*Source: adapted from [8].

## ESTIMATING SPENDING ON PANDEMIC PREPAREDNESS

An actionable approach to measuring country-level spending on preparedness would ideally possess the following characteristics. First, it must be comprehensive and capture all relevant expenditures from all sources – public, private, or international – related to prevention, detection, preparedness, and response. Second, it must be standardised, and common methodologies and definitions must be employed across countries to ensure consistency and comparability. Third, it must be transparent and readily make information on expenditures available to policymakers, stakeholders, and the public, enabling informed discussions and promoting accountability in the use of resources. Fourth, it must be integrated with existing health information or similar expenditure measurement systems so that data collection and reporting processes can be streamlined, reducing duplication of efforts, minimising reporting burdens, and enabling efficient data sharing and analysis. Fifth, it must facilitate international comparability, and the approach and methodologies must be aligned with global frameworks and standards that enable cross-country comparisons and the identification of best practices and areas for improvement. Lastly, it should not require such a significant allocation of resources that it becomes difficult for countries to implement it periodically. We propose three approaches that meet most of the above criteria: 1) using functional codes to classify preparedness-related public expenditures, 2) targeted surveys to capture preparedness-related public expenditures, and 3) extracting preparedness-related expenditures from health accounts.

### Preparedness-related functional codes

General government expenditures can be estimated by consolidating inputs from central, state, and local government accounting statements. In some cases, important government transactions occur through special or extrabudgetary funds (e.g. road or health funds). In others, quasi-fiscal operations are undertaken through the banking system (e.g. subsidised loans to state-owned enterprises). It is imperative, therefore, to identify all such transactions, quantify their magnitude, and include them within the consolidated total.

In an ideal scenario, all government expenditure transactions should be categorised across at least four dimensions – administrative responsibility (ministry or department), economic category (e.g. wages and salaries), function (such as health and education), and program (policy goals and objectives) [10]. This comprehensive coding would simplify the identification, tracking, and monitoring of spending across functions and economic categories and ensure that it aligns with specified objectives. Apart from its relative simplicity, this classification allows for a relatively simple determination of the recurrent expenditure outlays once the budget is established.

In most countries, health-related functional codes are commonly used to classify government transactions. We recommend enhancing this system by incorporating specific subcodes directly into the budget, allowing for the identification of preparedness-related spending. These subcodes can then be applied to payment orders, effectively isolating expenditures related to preparedness efforts. The initial phase of this initiative involves identifying all aspects of preparedness and precisely defining relevant categories or subcodes that align with the government’s objectives. Subsequently, the coding system must be seamlessly adjusted to integrate these new subcodes, ensuring compatibility with existing structures. Crucially, training pertinent personnel, including finance officers and administrators, in utilising these subcodes would be essential to maintain accurate and uniform coding practices across diverse departments.

Introducing a coding system with dedicated subcodes for preparedness-related spending brings numerous benefits, such as detailed tracking of funds allocated to preparedness efforts, enhanced transparency and accountability, and informed decision-making for policymakers with access to comprehensive spending information. It also facilitates the efficient allocation of resources by enabling authorities to identify areas where preparedness-related funds are most essential. However, developing and implementing such a new coding system, particularly one with additional subcodes, can be intricate and resource-intensive. Challenges may arise due to associated costs, including modifying existing systems, training personnel, and ensuring compatibility.

### Targeted surveys

A targeted survey-based approach can help estimate government spending on pandemic preparedness by directly engaging relevant stakeholders, such as health departments and emergency management agencies. The survey can provide specific and accurate data by tailoring questions to capture expenditures related to infrastructure, human resources, training, research, and stockpiling of medical supplies. This approach enables a more comprehensive understanding of budget allocations and allows for a nuanced analysis, ensuring that resources are effectively utilised in preparing for future pandemics. One such survey instrument is the specially designed Health Security Financing Assessment Tool (HSFAT), which maps spending on the JEE technical areas [11].

Developed by the World Bank to help governments improve pandemic preparedness and response and enable sustainable financing for health security, HSFAT is aligned with International Health Regulations and complements annual reporting, after-action reviews, simulation exercises, and the JEE. The HSFAT methodology makes a distinction between ‘health security specific’ activities within the JEE technical areas domains, such as those associated with strengthening specialised laboratories, food safety, emergency operations and health security sensitive activities, which include spending on workforce development and wildlife veterinary services that have an impact on health security. In the final counting, the HSFAT methodology attributes all spending on health-security-specific activities to strengthening preparedness but only a portion, determined by expert opinion, of spending on health-security-sensitive activities to strengthening preparedness. Three countries – Vietnam, Pakistan and Indonesia – have employed the HSFAT to estimate preparedness spending.

A survey-based method for estimating government spending on pandemic preparedness offers a significant advantage by thoroughly examining particular spending facets, yielding detailed insights into resource allocation across diverse activities and sectors. Moreover, these surveys can be tailored to collect up-to-date information. Conversely, the main challenges associated with surveys include ensuring data accuracy and reliability, addressing incomplete information, and navigating the considerable costs and time commitments involved in their implementation.

### Health accounts

The system of health accounts (SHAs) is a systematic and comprehensive framework for tracking and analysing health spending within a country. It helps answer three important questions – what is being spent, where is it being spent, and who is paying. SHAs organise the spending data according to three core dimensions: financing schemes, i.e. the main types of financing arrangements through which people receive health care, health care providers, i.e. actors that deliver health care, and health care functions, i.e. types of health services or goods that are produced, such as curative care, rehabilitative care, long term care, ancillary services, medical goods, preventive care, and governance and administration. The health expenditure data are presented as matrices that provide information on various intersections, such as financing scheme-to-provider, provider-to-function, financing scheme-to-function, etc. [12]. Adopted by more than 100 countries as an essential financing tool for managing the health system, this common classification and accounting framework for measuring health spending allows for easy cross-country and cross-time comparisons.

To assess the feasibility and simplicity of retracing preparedness spending from SHAs by the JEE technical elements, we conducted case studies in four countries – Bangladesh, Indonesia, Pakistan, and Vietnam. Each country had recently evaluated preparedness for strengthening spending through annual reviews of budgetary expenditures (Bangladesh) or the HSFAT tool (Indonesia, Pakistan, and Vietnam). We inferred preparedness spending from these countries’ financing-to-functions matrix of health accounts and compared the results with those reported in the countries’ evaluations. This comparison allowed us to make informed judgments about the practicality and applicability of using health accounts to ascertain preparedness spending.

We would expect spending recorded under immunisation and surveillance functional sub-categories of SHAs to correspond closely with JEE technical areas related to these two functions. Likewise, we would expect some of the spending recorded under the governance and health system administration functions of SHAs to encompass preparedness spending as recorded in such JEE technical areas as legal documentation, International Health Regulations coordination, food safety, emergency response operations, etc. Our findings broadly support our first impressions. We found that between 60–70% of total spending by SHAs function corresponded to the JEE technical areas of immunisation, surveillance, and risk communication. Likewise, we found that 10–12% of spending recorded under governance and health system administration corresponded with several other JEE technical areas. Less than 2% of spending recorded under all other SHAs functional categories could be allocated to preparedness.

However, we could not extract granular preparedness spending data from health accounts for several reasons. First, the SHAs functions are not fully aligned with the boundaries of preparedness as defined by the JEE technical areas. For example, SHAs Classification of Health Care Functions 6.2 covers all immunisations, while the JEE immunisation target is defined as a national vaccine delivery system with nationwide reach, effective distribution, easy access for marginalised populations, adequate cold chain, and ongoing quality control that can respond to new disease threats. Likewise, SHAs functional category Classification of Health Care Functions 6.5. covers epidemiological surveillance and risk and disease control programs, while the JEE surveillance relates to indicator- and event-based surveillance systems that can detect events of significance for public health and health security and are measured by surveillance for at least three core syndromes indicative of potential public health emergencies.

Second, a quick review of SHAs of several countries revealed that most do not provide disaggregated information on most functional categories. For example, surveillance and risk communication are lumped together, making it almost impossible to extract disaggregated information on either of the two JEE technical areas. Third, we identified huge variations in the SHAs in the proportion of spending according to functional categories. In Bangladesh, for instance, nearly half of all health spending was allocated to medical goods, and pharmacies accounted for 46% of total health expenditure in the country. Similarly, Indonesia reported that 72% of its health spending was dedicated to curative care, with hospitals and ambulatory care providers accounting for 70% of all health spending. However, allocating funds to preventive care is paramount for preparedness, which may not be fully captured, especially if some preventive care services are provided within hospital and ambulatory care settings. These specific features of the respective health systems hinder standardisation and make establishing clear guidelines for identifying preparedness spending from health accounts challenging.

Despite these challenges, we believe there is significant value in aligning the assessment of preparedness expenditures in the health sector with health accounts. This alignment is crucial as a substantial portion of preparedness spending occurs within the health sector. Many countries have already gained experience with health accounts and have invested in enhancing their capabilities to monitor and trace the flow of funds in the health domain. While extracting preparedness spending data from SHAs would necessitate some functional definitions and boundary adjustments, the undertaking would prove highly beneficial. Importantly, it would prevent redundant efforts, alleviate the reporting burden on governmental agencies and information-providing partners, and ensure that estimates of key variables remain closely aligned with actual spending. Furthermore, completing the SHAs would present a relatively swift and cost-effective method for retroactively tracking data on preparedness spending.

We found no equivalent instrument to health accounts for assessing spending related to animal health preparedness. The most comprehensive attempt to estimate spending on crucial aspects of preparedness was the 2009 study commissioned by the World Organization for Animal Health [13]. This study aimed to calculate the costs of national prevention systems for animal diseases and zoonoses in nine countries. Serving as a functional foundation, this study provides a basis for standardising a framework for collecting data on preparedness spending in the animal health sector.

Regularly updated in accordance with guidance from the World Organization for Animal Health’s Terrestrial and Aquatic Animal Health Codes [14], which establish standards for enhancing animal health, welfare, and veterinary public health globally, the definitions, boundaries, and functional classifications in the 2009 World Organization for Animal Health study offer a practical approach to measuring and tracking spending on preparedness in the animal health sector.

## CONCLUSIONS

Despite the growing demand for and accessibility of financing for preparedness, there is a notable lack of clarity regarding the actual expenditure on preparedness in many countries. It is imperative for development partners and countries to collaboratively establish straightforward, actionable, and cost-effective methods for measuring preparedness spending. This collaborative effort is essential for monitoring purposes and enhancing efficiency in allocating resources toward preparedness initiatives. By establishing transparent and efficient measurement mechanisms, stakeholders can ensure that financial resources are utilised optimally, contributing to a more effective and responsive preparedness infrastructure.
